# Ultrasound-Guided Intra-articular Prolotherapy Versus Corticosteroid Injection for Sacroiliac Joint Dysfunction: A Prospective Randomized Controlled Trial

**DOI:** 10.7759/cureus.117276

**Published:** 2026-10-01

**Authors:** Ruth Kikon, Anil K Gupta, Ganesh Yadav, Dileep Kumar, Sandeep K Gupt, Joe Antony, Jay Vardhan Kumar, Neelam Kumari

**Affiliations:** 1 Physical Medicine and Rehabilitation, King George's Medical University, Lucknow, IND

**Keywords:** chronic low back pain, corticosteroid injection, numeric pain rating scale, oswestry disability index, prolotherapy, sacroiliac joint dysfunction, ultrasound-guided injection

## Abstract

Introduction: Sacroiliac (SI) joint dysfunction is an important cause of chronic low back pain and may be difficult to diagnose and treat. Corticosteroid injection is commonly used for short-term symptom relief, whereas the comparative durability of prolotherapy remains uncertain. This study aimed to compare ultrasound-guided intra-articular prolotherapy with corticosteroid injection in patients with SI joint dysfunction, with pain intensity and functional disability assessed over a 12-week follow-up period.

Methods: This prospective randomized controlled trial included 38 patients with SI joint dysfunction. Following a diagnostic intra-articular lignocaine block, patients demonstrating at least 50% pain reduction were randomized in a 1:1 ratio to receive ultrasound-guided intra-articular triamcinolone acetonide (40 mg) or 25% dextrose prolotherapy. All participants received an identical supervised physiotherapy program. Pain intensity and functional disability were assessed using the Numeric Pain Rating Scale (NPRS) and Oswestry Disability Index (ODI), respectively, at baseline, immediately after intervention, and at 4 and 12 weeks.

Results: Nineteen patients were allocated to each group, and all participants completed follow-up. Baseline NPRS scores were comparable between the corticosteroid and prolotherapy groups (8.47 ± 0.77 vs. 8.84 ± 0.38; p=0.1115). Immediately after intervention, NPRS scores decreased substantially in both groups (1.47 ± 0.84 vs. 1.11 ± 0.32; p=0.1044). At 12 weeks, the mean NPRS score was significantly lower in the prolotherapy group than in the corticosteroid group (1.11 ± 0.32 vs. 4.05 ± 1.55; p<0.0001). The mean ODI score at 12 weeks was also significantly lower in the prolotherapy group (8.53 ± 3.19 vs. 21.42 ± 6.82; p<0.0001). No major procedure-related complications or serious adverse events were reported.

Conclusion: Both ultrasound-guided intra-articular prolotherapy and corticosteroid injection improved pain and functional disability in patients with sacroiliac joint dysfunction. Prolotherapy was associated with lower pain and disability scores at 4 and 12 weeks compared with corticosteroid injection.

## Introduction

Sacroiliac (SI) joint dysfunction is increasingly recognized as an important cause of low back pain, accounting for approximately 15-30% of cases in carefully selected patient populations [1,2]. Despite its clinical significance, SI joint dysfunction remains underdiagnosed because its clinical presentation frequently overlaps with disorders of the lumbar spine and hip, while the complex anatomy and biomechanics of the joint make accurate diagnosis challenging [3,4]. SI-region pain may arise from intra-articular or extra-articular structures. Important alternative diagnoses include inflammatory sacroiliitis, infection, fracture, hip disorders, lumbar spine disorders, and myofascial pain. Accurate clinical assessment is therefore essential to distinguish mechanical sacroiliac joint dysfunction from other conditions that may present with similar symptoms [3-5].

The sacroiliac joint is a strong articulation connecting the sacrum and ilium and functions as a major load-transmitting structure between the spine and lower extremities while allowing limited movement [3]. Its complex sensory innervation contributes to variable pain patterns that may involve the low back, buttock, groin, and posterior thigh and can mimic lumbar disc or facet-mediated pain [1,3,5]. Consequently, SI joint pain may present with symptoms that overlap considerably with other causes of low back and lower-extremity pain [5]. A clear understanding of the anatomical and neurophysiological basis of SI joint pain is therefore important for establishing an accurate diagnosis and selecting an appropriate treatment strategy.

Management of SI joint dysfunction is primarily conservative and aims to reduce pain and restore functional capacity. Patients who do not respond adequately to conservative measures may be considered for interventional procedures, including image-guided intra-articular injections and radiofrequency-based interventions [4,6,7]. Radiofrequency techniques have been investigated as treatment options for persistent SI joint pain, while image-guided injection techniques have been developed to improve procedural accuracy [7,8]. Ultrasound guidance has also been evaluated as an alternative to fluoroscopic guidance for SI joint injections [9], offering the potential advantages of real-time visualization of relevant anatomical structures without ionizing radiation.

Accurate diagnosis of SI joint dysfunction remains challenging because no single clinical test is sufficiently diagnostic. A combination of clinical history, physical examination, multiple SI joint provocation tests, and confirmatory diagnostic injections is generally used in clinical practice [6,8,10]. When diagnostic uncertainty persists, image-guided intra-articular local anesthetic injection may provide additional diagnostic confirmation when substantial pain relief is obtained following the block [8,10]. These approaches are particularly relevant because appropriate identification of the SI joint as the primary pain generator is essential before proceeding with targeted interventional treatment.

Among interventional treatment options, corticosteroid injections and prolotherapy have gained considerable attention. Prolotherapy uses hypertonic dextrose solutions with the proposed aim of stimulating tissue healing and improving joint stability. Hoffman and Agnish [10] and Mitchell and Barnard [11] reported favorable functional outcomes following SI joint prolotherapy in patients with SI joint instability. Corticosteroid injections provide anti-inflammatory effects and have demonstrated clinical benefit in patients with SI joint dysfunction. Lee et al. also reported beneficial effects of intra-articular prolotherapy in patients with SI joint pain [12]. Buijs et al. highlighted the potential contribution of the SI joint to pain syndromes that may mimic other spinal or lower-extremity conditions [13]. Collectively, these findings support the use of injection-based interventions as potential treatment options for appropriately selected patients with SI joint dysfunction.

Comparative evidence regarding prolotherapy and corticosteroid treatment has also begun to emerge. Kim et al. conducted a randomized controlled trial comparing intra-articular prolotherapy with steroid injection for SI joint pain and reported improvement following both interventions, with more sustained benefit observed following prolotherapy [14]. A systematic review comparing prolotherapy with corticosteroid treatment for SI joint dysfunction has similarly suggested potential advantages of prolotherapy, although the available evidence remains limited [6]. These findings raise the possibility that prolotherapy may provide a more sustained therapeutic effect than corticosteroid injection in selected patients with SI joint dysfunction. However, variations in study design, injection techniques, patient characteristics, outcome measures, and follow-up duration across the available literature make direct comparisons difficult.

Although both corticosteroid injection and prolotherapy have demonstrated clinical benefit [6,10,12,14], direct comparative evidence between these two interventions remains scarce. In particular, evidence directly comparing ultrasound-guided intra-articular prolotherapy with ultrasound-guided corticosteroid injection is limited. Consequently, the optimal injection-based treatment for SI joint dysfunction has not been clearly established. A direct comparison of these interventions using a standardized ultrasound-guided intra-articular technique may therefore provide clinically relevant evidence regarding their relative efficacy and durability of treatment response.

Pain intensity and functional disability are important outcomes when evaluating treatment response in patients with SI joint dysfunction. Evidence-based diagnostic approaches emphasize the use of clusters of provocation tests rather than reliance on an individual maneuver [15]. The Numeric Pain Rating Scale (NPRS) is a validated measure of pain intensity [16]. The Oswestry Disability Index (ODI) is a widely used patient-reported measure of disability associated with low back pain [17]. Assessment of both pain intensity and functional disability provides a broader evaluation of treatment effectiveness by considering not only symptomatic improvement but also the patient's ability to perform daily activities.

Therefore, the present prospective randomized controlled study was undertaken to compare ultrasound-guided intra-articular prolotherapy with corticosteroid injection in patients with sacroiliac joint dysfunction, with pain intensity and functional disability as the primary outcomes, assessed using the Numeric Pain Rating Scale (NPRS) and Oswestry Disability Index (ODI), respectively, at baseline, immediately after the intervention, and at 4 and 12 weeks. By directly comparing these two injection-based interventions, using ultrasound guidance, this study aimed to contribute to the limited comparative evidence regarding the management of SI joint dysfunction and to determine whether prolotherapy provides greater or more sustained clinical benefit than corticosteroid injection. The findings of this study may help inform evidence-based treatment strategies for patients with SI joint pain.

## Materials and methods

Study design and participants

This was a single-centre, prospective, patient-blinded, randomized controlled trial that included 38 participants with sacroiliac joint dysfunction. Following a positive diagnostic intra-articular lignocaine block, eligible participants were randomized to receive either an ultrasound-guided intra-articular triamcinolone acetonide injection or 25% dextrose prolotherapy. Participants were not informed whether they had received corticosteroid injection or prolotherapy. Clinical outcomes were assessed using the Numeric Pain Rating Scale and Oswestry Disability Index [16,17] at baseline, immediately after the intervention, and at 4 and 12 weeks following treatment.

Participants aged 18 to 60 years of either sex were considered eligible for inclusion. Additional inclusion criteria were pain localized over the sacroiliac joint, a diagnosis of sacroiliac joint dysfunction based on clinical assessment and appropriate diagnostic criteria, including clinical provocation testing and confirmation with a diagnostic intra-articular lignocaine block [10], and low back pain predominantly below the L5 vertebral level. All participants were required to provide written informed consent before enrollment.

Patients were excluded if they were receiving anticoagulant therapy or had uncontrolled diabetes mellitus, known hypersensitivity to lignocaine, local or systemic infection, skin lesions at the injection site, poor general health, or psychiatric illness that could affect participation in the study. Patients with a history of systemic or local corticosteroid injection within the preceding three months were also excluded. Additional exclusion criteria included hip joint pathology, spinal or pelvic deformity, infective or neoplastic pathology involving the spine, and spondyloarthropathies.

Sample size

The sample size was calculated using a formula for comparison of two proportions based on the study by Kim et al. [14]. Assuming a two-sided alpha error of 5% and a study power of 80%, the required sample size was calculated to be 38 participants. Accordingly, 38 participants were recruited and randomized equally between the corticosteroid and prolotherapy groups.

Randomization

Participants who fulfilled the eligibility criteria and demonstrated at least 50% pain relief following the diagnostic intra-articular lignocaine block were randomly allocated in a 1:1 ratio to either the corticosteroid group (Group A) or the prolotherapy group (Group B) using a computer-generated randomization sequence. A total of 38 participants were randomized, with 19 participants allocated to each treatment group.

Baseline assessment

A detailed medical history and physical examination were performed for all participants before intervention. Baseline laboratory investigations, including complete hemogram, erythrocyte sedimentation rate (ESR), and C-reactive protein (CRP), were performed to exclude inflammatory causes of low back pain. Radiographic evaluation included anteroposterior and lateral radiographs of the lumbosacral spine and an anteroposterior radiograph of the pelvis including both hips. Magnetic resonance imaging was performed in participants with suspected early spondyloarthropathy.

Pain intensity and functional disability were assessed at baseline using the NPRS and ODI, respectively [16,17]. These measures were subsequently used to evaluate changes in pain intensity and functional disability following the intervention.

Intervention procedure

Following written informed consent, participants were transferred to the procedure room. Baseline cardiorespiratory parameters were recorded using a multiparameter monitor, and intravenous access was established. Each participant was positioned prone, and the sacroiliac joint was identified under ultrasound guidance. After appropriate skin preparation and strict aseptic precautions, a needle was introduced into the inferior aspect of the sacroiliac joint, approximately 1 cm distal to the inferior joint margin (Figure 1).

**Figure 1:**
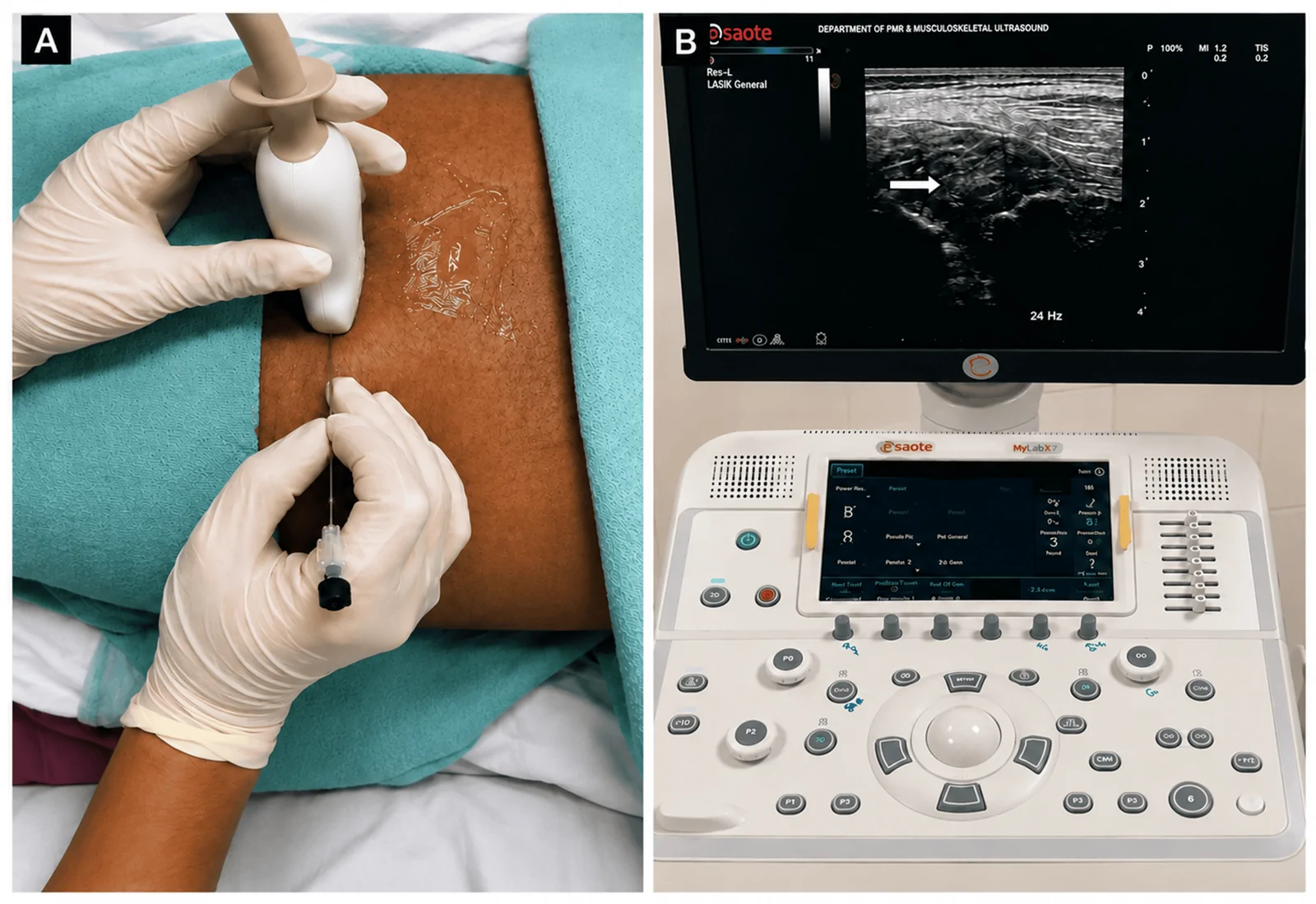
Ultrasound-guided intra-articular sacroiliac joint injection performed in the Department of Physical Medicine and Rehabilitation, King George's Medical University, Lucknow (A) Needle placement after identifying the inferior aspect of the sacroiliac (SI) joint. (B) Ultrasound image demonstrating drug entry into the SI joint; the white arrow indicates the site of drug entry.

A diagnostic intra-articular block was initially performed using 1 mL of 2% lignocaine. Participants who demonstrated at least a 50% reduction in pain following the diagnostic block were considered to have a positive response and subsequently received the allocated study intervention.

Participants assigned to Group A, the corticosteroid group, received an ultrasound-guided intra-articular injection consisting of 1 mL of 2% lignocaine and 1 mL containing 40 mg of triamcinolone acetonide. Participants assigned to Group B, the prolotherapy group, received an ultrasound-guided intra-articular injection consisting of 1 mL of 25% dextrose and 1 mL of 2% lignocaine. The injection procedure was performed using the same ultrasound-guided approach in both groups.

Rehabilitation protocol

All participants underwent an identical supervised physiotherapy program following the intervention. The rehabilitation program consisted of core stabilization and strengthening exercises performed for 20 minutes per session, three sessions per week, and continued for 12 weeks. The same rehabilitation protocol was provided to participants in both treatment groups to maintain consistency in post-intervention care.

Outcome measures

The primary outcome measures were pain intensity and functional disability, assessed using the NPRS and ODI, respectively [16,17]. Outcome assessments were performed at baseline before the intervention, immediately after the procedure, and at 4 and 12 weeks following the intervention. Changes in NPRS and ODI scores were evaluated over the follow-up period to compare the clinical responses to ultrasound-guided intra-articular corticosteroid injection and prolotherapy.

Statistical analysis

Data were entered into Microsoft Excel (Microsoft Corporation, Redmond, USA) and analyzed using IBM SPSS Statistics for Windows, version 26.0 (IBM Corp., Armonk, USA). Statistical analysis was performed under the supervision of a statistician from the Clinical Epidemiology Unit. Continuous variables were expressed as means ± standard deviations or medians with interquartile ranges, as appropriate, whereas categorical variables were presented as frequencies and percentages.

Between-group and within-group comparisons were performed using appropriate parametric or non-parametric statistical tests according to the distribution and characteristics of the data. A two-tailed p-value of <0.05 was considered statistically significant.

Ethical considerations

The study protocol was approved by the Institutional Ethics Committee before commencement of the study. The study was conducted in accordance with the ethical principles outlined in the Declaration of Helsinki. Written informed consent was obtained from all participants before enrollment in the study.

The trial was prospectively registered with the Clinical Trials Registry-India (CTRI; registration number CTRI/2024/05/067511).

## Results

Participant recruitment and baseline characteristics

A total of 38 patients who met the eligibility criteria were enrolled and randomized in a 1:1 ratio to the corticosteroid or prolotherapy group. Nineteen patients were allocated to each group. All participants completed the scheduled 12-week follow-up assessments, and all 38 participants were included in the final analysis (Figure 2).

**Figure 2:**
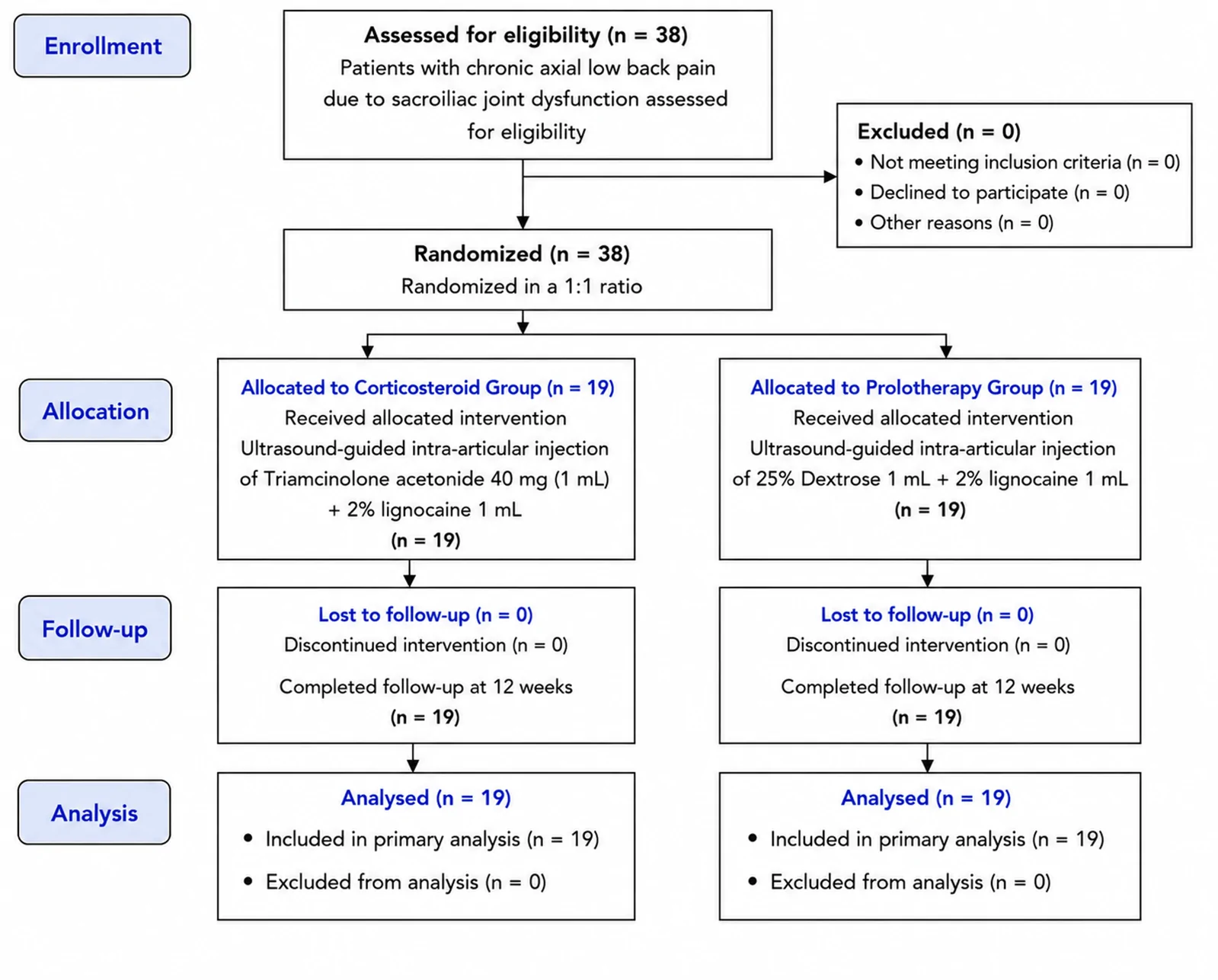
CONSORT flow diagram CONSORT: Consolidated Standards of Reporting Trials

The mean age of the study population was 33.57 ± 13.46 years. Female patients constituted 76% of the study population, while male patients accounted for 24% (Table 1). The corticosteroid and prolotherapy groups were comparable with respect to age, body mass index (BMI), duration of symptoms, and baseline pain and disability scores. No statistically significant between-group differences were observed at baseline, indicating comparability of the randomized groups.

**Table 1:** Baseline demographic and clinical characteristics Continuous variables are presented as means ± SDs and were compared using the independent-samples t-test. Categorical variables are presented as n (%) and were compared using the chi-square test or Fisher’s exact test, as appropriate.

Variable	Corticosteroid (n=19)	Prolotherapy (n=19)	Test statistic (df)	p-value
Age (years)	35.84 ± 15.75	31.32 ± 10.58	t = 1.038 (36)	0.3054
BMI (kg/m²)	22.85 ± 4.41	24.31 ± 4.62	t = −0.996 (36)	0.3274
Duration of pain (months)	15.53 ± 10.56	12.74 ± 8.23	t = 0.908 (36)	0.3698
Female, n (%)	29 (76%), overall	—	—	—

Pain outcome

Pain intensity was assessed using the Numeric Pain Rating Scale. Baseline NPRS scores were comparable between the corticosteroid and prolotherapy groups (8.474 ± 0.772 vs. 8.842 ± 0.375; p=0.1115; mean difference, −0.37; 95% CI, −0.77 to 0.04).

Immediately after the intervention, both groups demonstrated marked reductions in pain intensity. The mean NPRS scores decreased to 1.474 ± 0.841 in the corticosteroid group and 1.105 ± 0.315 in the prolotherapy group. The between-group difference was not statistically significant (p=0.1044; mean difference, 0.37; 95% CI, −0.06 to 0.80).

At four weeks, the mean NPRS score was significantly lower in the prolotherapy group than in the corticosteroid group (1.105 ± 0.315 vs. 2.421 ± 1.216; p<0.0001; mean difference, 1.32; 95% CI, 0.72-1.92). At 12 weeks, the mean NPRS score remained substantially lower in the prolotherapy group than in the corticosteroid group (1.105 ± 0.315 vs. 4.053 ± 1.545; p<0.0001; mean difference, 2.95; 95% CI, 2.19-3.70) (Table 2; Figure 3).

**Table 2:** Comparison of Numeric Pain Rating Scale (NPRS) scores between the corticosteroid (Group A) and prolotherapy (Group B) groups at baseline, immediately after intervention, and at 4 and 12 weeks of follow-up Data are presented as means ± standard deviations (SDs). Between-group comparisons were performed using the independent-samples t-test, and changes across repeated follow-up measurements were assessed using repeated-measures analysis. A two-tailed p-value <0.05 was considered statistically significant.

Follow-up	Group A	Group B	p-value
Baseline	8.474 ± 0.772	8.842 ± 0.375	0.1115
Immediate	1.474 ± 0.841	1.105 ± 0.315	0.1044
4 weeks	2.421 ± 1.216	1.105 ± 0.315	<0.0001
12 weeks	4.053 ± 1.545	1.105 ± 0.315	<0.0001

**Figure 3:**
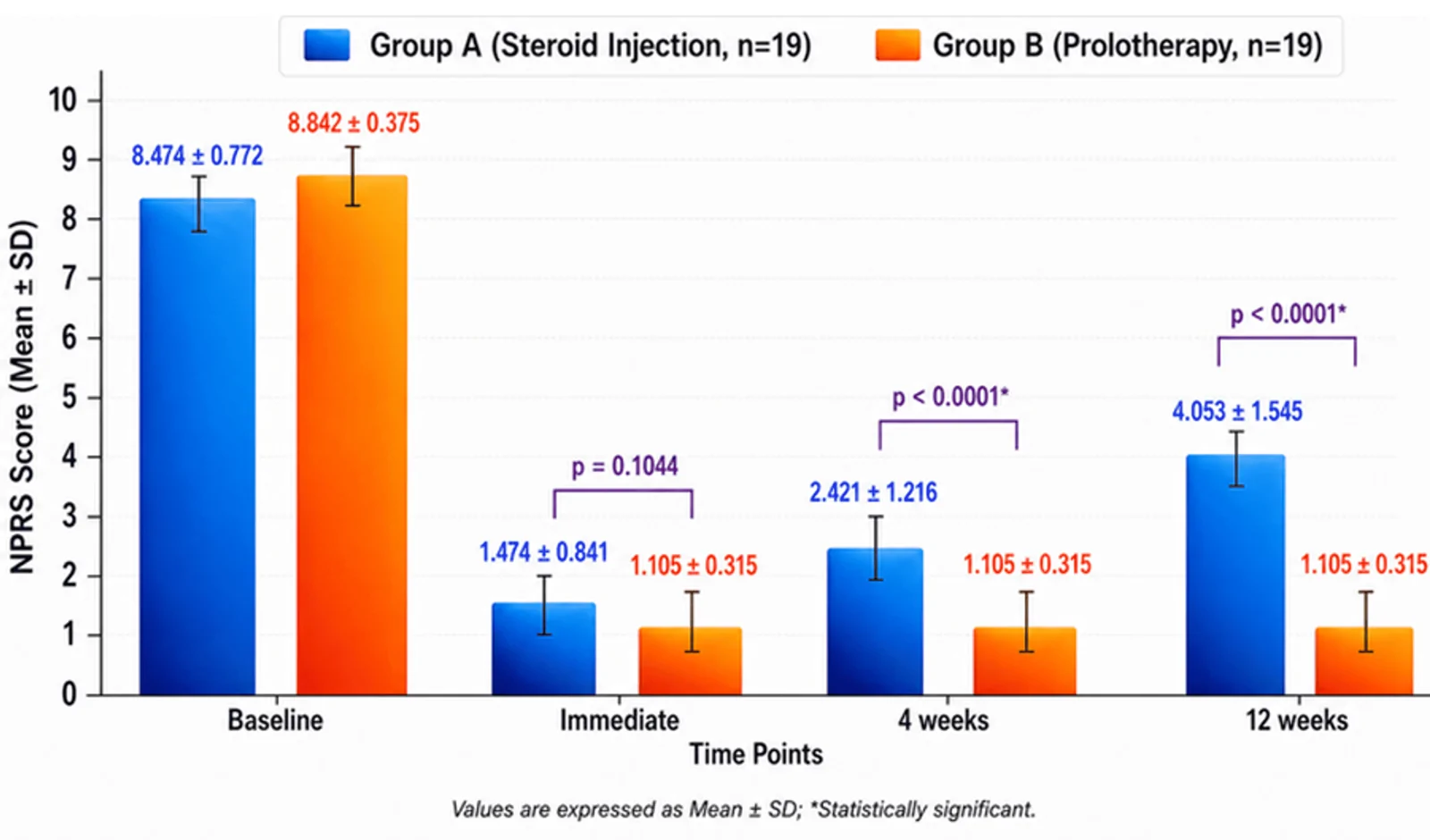
Comparison of Numeric Pain Rating Scale (NPRS) scores between Group A and Group B at different time points Image created using Microsoft Excel (Microsoft Corporation, Redmond, USA).

Within-group analysis demonstrated a significant reduction in the NPRS score from baseline to 12 weeks in both the corticosteroid and prolotherapy groups (p<0.0001 for both groups) (Table 2; Figure 3).

Functional outcome

Functional disability was assessed using the Oswestry Disability Index. At baseline, the mean ODI score was 34.11 ± 4.37 in the corticosteroid group and 34.68 ± 3.59 in the prolotherapy group. The difference between the groups was not statistically significant (p = 0.6582; mean difference, −0.57; 95% CI, −3.20 to 2.06), indicating comparable baseline functional disability between the two groups (Table 3; Figure 4).

**Table 3:** Comparison of Oswestry Disability Index (ODI) scores between the corticosteroid (Group A) and prolotherapy (Group B) groups at baseline and at 4 and 12 weeks of follow-up Data are presented as means ± standard deviations (SD). Between-group comparisons were performed using appropriate statistical tests based on data distribution. Changes in ODI scores across follow-up time points were assessed using repeated-measures analysis. A two-tailed p-value <0.05 was considered statistically significant.

Follow-up	Group A	Group B	P value
Baseline	34.11 ± 4.37	34.68 ± 3.59	0.6582
4 weeks	18.95 ± 6.51	10.05 ± 3.44	<0.0001
12 weeks	21.42 ± 6.82	8.53 ± 3.19	<0.0001

**Figure 4:**
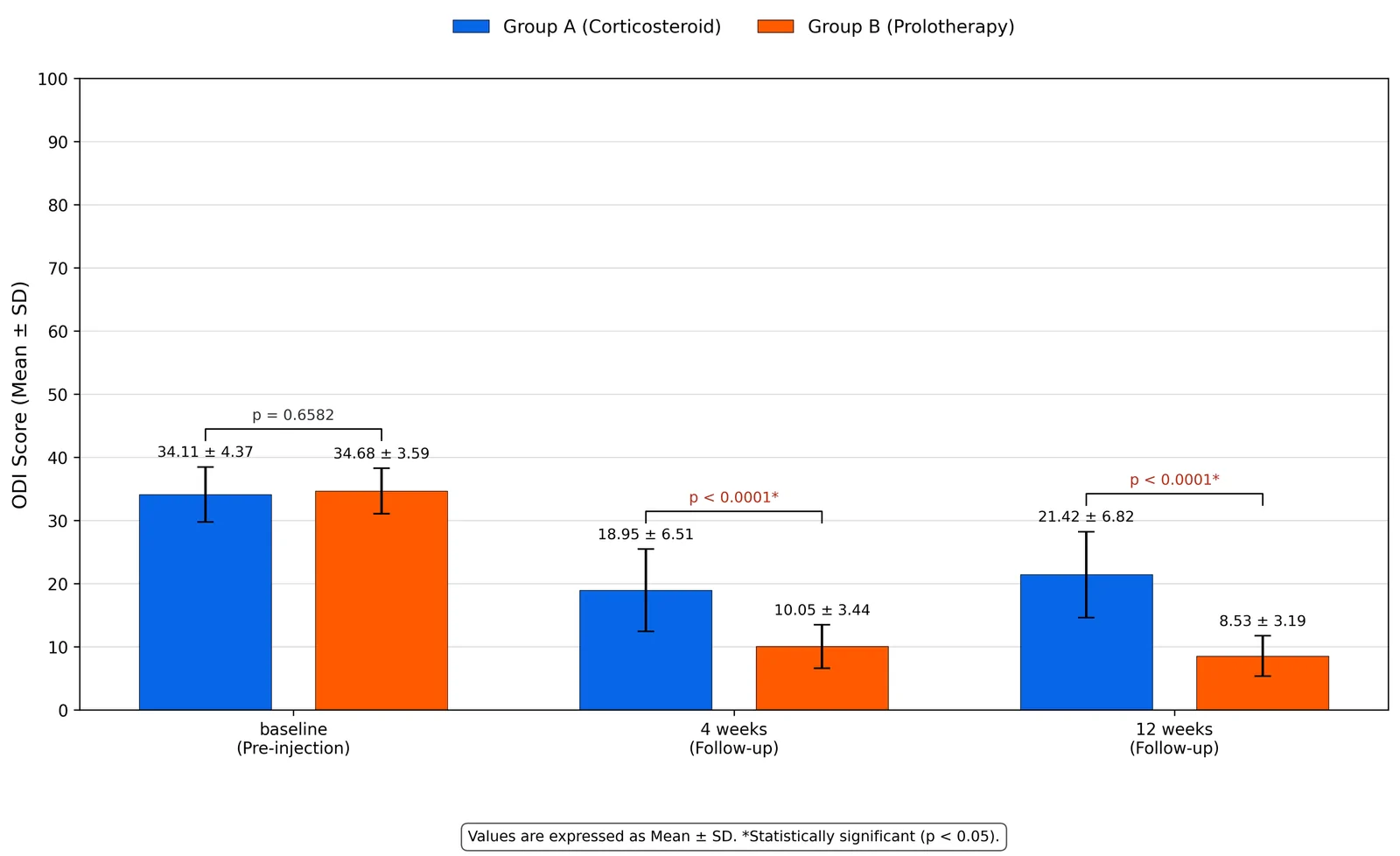
Comparison of Oswestry Disability Index (ODI) scores between Group A and Group B at different time points Image created using Microsoft Excel (Microsoft Corporation, Redmond, USA).

At four weeks, both groups demonstrated improvement in functional disability, with the mean ODI score decreasing to 18.95 ± 6.510 in the corticosteroid group and 10.05 ± 3.440 in the prolotherapy group. The prolotherapy group demonstrated significantly lower ODI scores than the corticosteroid group, with a mean difference of 8.90 (95% CI, 5.44 to 12.36; p < 0.0001). At 12 weeks, the mean ODI score was 21.42 ± 6.818 in the corticosteroid group and 8.526 ± 3.186 in the prolotherapy group. The between-group difference remained statistically significant in favor of prolotherapy, with a mean difference of 12.89 (95% CI, 9.34 to 16.45; p < 0.0001) (Table 3; Figure 4). The ODI results therefore demonstrated a greater and more sustained improvement in functional disability following prolotherapy compared with corticosteroid injection.

Safety

No major procedure-related complications or serious adverse events were reported during the study period. Both ultrasound-guided intra-articular corticosteroid injection and prolotherapy were well tolerated by the participants throughout the 12-week follow-up period. No participants discontinued the intervention or were lost to follow-up, and all 38 participants completed the scheduled assessments.

## Discussion

The present prospective randomized controlled trial compared the clinical efficacy of ultrasound-guided intra-articular corticosteroid injection and prolotherapy in patients with sacroiliac joint dysfunction. Both randomized groups showed improvement from baseline in pain intensity and functional disability during the 12-week follow-up period. The comparative finding was lower pain and disability scores in the prolotherapy group at 4 and 12 weeks. Immediate post-procedural pain relief was comparable between the groups. These findings indicate a between-group difference in pain and functional outcomes during follow-up, with lower scores observed in the prolotherapy group at 4 and 12 weeks.

The baseline demographic and clinical characteristics were comparable between the two groups, supporting successful randomization and reducing the likelihood that baseline differences substantially influenced the observed outcomes. The present study included 76% female and 24% male participants. Previous literature has reported a female predominance among patients with SI joint dysfunction. Several mechanisms have been proposed to contribute to sex-related differences in susceptibility, including differences in pelvic anatomy, ligamentous laxity, hormonal influences, and neuromuscular factors. However, the female predominance observed in the present study may also reflect the characteristics of the study population and recruitment setting.

One of the principal findings of the present study was the sustained reduction in pain following prolotherapy. Both interventions produced substantial immediate pain relief; however, pain scores in the corticosteroid group increased during subsequent follow-up, whereas the prolotherapy group maintained significantly lower NPRS scores at 4 and 12 weeks. These findings are consistent with the randomized controlled trial by Kim et al. [14], which reported more sustained pain relief following prolotherapy compared with corticosteroid injection. Similar sustained analgesic effects following dextrose prolotherapy have also been reported by Lee et al. [12]. In contrast, the clinical benefits reported for corticosteroid injections for SI joint pain have generally been more pronounced in the short term. The pattern observed in the present study therefore suggests a potentially greater durability of response following prolotherapy.

The greater persistence of pain reduction observed following prolotherapy may have important clinical implications. While corticosteroid injections may provide effective short-term symptom relief through their anti-inflammatory effects, the progressive increase in pain scores observed during follow-up in the corticosteroid group suggests that the therapeutic effect may diminish over time. Prolotherapy, in contrast, may have effects that persist beyond the immediate post-injection period. Although the precise mechanisms responsible for this difference remain uncertain, the temporal pattern observed in the present study is consistent with the possibility that prolotherapy may offer a more durable therapeutic response in selected patients with SI joint dysfunction.

Functional outcomes demonstrated a similar pattern. Although both groups showed significant improvement in ODI scores, the magnitude and persistence of functional improvement were greater in the prolotherapy group. These findings are consistent with previous studies by Kim et al. [14] and Hoffman and Agnish [10], which reported favorable functional outcomes following prolotherapy. The sustained reduction in disability observed in the present study may indicate that the benefits of prolotherapy extend beyond short-term analgesia and may contribute to improved functional capacity and participation in daily activities. The concurrent improvement in pain and disability outcomes further supports the clinical relevance of the treatment response observed in the prolotherapy group.

The clinical response to prolotherapy may be related to the proposed biological effects of hypertonic dextrose. Hypothesized mechanisms of dextrose prolotherapy include modulation of local nociception and induction of connective-tissue responses, including fibroblast activity and collagen synthesis, which may contribute to tissue remodeling. However, these mechanisms were not directly assessed in the present trial, and the study cannot determine the biological mechanism underlying the observed clinical outcomes. In contrast, corticosteroids primarily exert anti-inflammatory effects and are generally considered to provide symptomatic relief rather than directly promoting tissue remodeling. These differences in the proposed mechanisms of action may partly explain the more sustained clinical improvement observed following prolotherapy in the present study. However, these mechanisms remain theoretical in the context of the present clinical findings, and further studies incorporating imaging, biomechanical, or other objective assessments would be required to directly demonstrate structural changes associated with prolotherapy and to establish a causal relationship between tissue remodeling and sustained clinical improvement.

The findings of the present study may have potential relevance to rehabilitation practice and the interventional management of sacroiliac joint dysfunction. Ultrasound-guided prolotherapy may be considered as a treatment option for patients with persistent symptoms despite appropriate conservative management. The sustained improvements in pain and functional disability observed in the prolotherapy group may be particularly relevant when longer-lasting symptom control is desired. Ultrasound guidance may further facilitate accurate needle placement and visualization of the target region during injection. Nevertheless, the selection of prolotherapy or corticosteroid injection should be individualized according to patient characteristics, clinical presentation, treatment goals, and clinician expertise.

The present study has several strengths. The prospective randomized controlled design allowed direct comparison of the two interventions while reducing the potential influence of selection bias and baseline differences. The use of an ultrasound-guided injection technique provided a standardized approach to intervention, while the standardized rehabilitation program helped minimize the potential influence of differences in post-procedural rehabilitation. In addition, the use of validated patient-reported outcome measures, including the NPRS and ODI, allowed assessment of both pain intensity and functional disability. Evaluating these complementary outcomes provided a broader assessment of the clinical response to treatment than pain assessment alone.

Some limitations should be considered when interpreting the findings. The study was conducted at a single center and included a relatively small sample size, which may limit the generalizability of the results to larger and more diverse patient populations. In addition, the follow-up period was limited to 12 weeks, and therefore, the present study cannot establish the long-term durability of the observed treatment effects. Although prolotherapy demonstrated more sustained improvements during the study period, these findings should not be interpreted as definitive evidence of long-term superiority over corticosteroid injection. Furthermore, the mechanisms underlying the observed differences in treatment response were not directly evaluated through imaging or biomechanical assessments. Larger multicenter randomized controlled trials with longer follow-up are therefore warranted to confirm the observed benefits, determine their long-term durability, and further evaluate the comparative effectiveness of prolotherapy and corticosteroid injection.

Overall, the findings of the present study indicate that both ultrasound-guided intra-articular corticosteroid injection and prolotherapy can improve pain intensity and functional disability in patients with sacroiliac joint dysfunction. However, prolotherapy was associated with greater and more sustained improvements in NPRS and ODI scores over the 12-week follow-up period. These findings suggest that prolotherapy may provide a more sustained medium-term clinical benefit than corticosteroid injection in selected patients with sacroiliac joint dysfunction. Further adequately powered multicenter studies with longer follow-ups are needed to determine whether the observed benefits are maintained over the longer term and to establish the comparative effectiveness of these interventions in the management of sacroiliac joint dysfunction.

## Conclusions

Ultrasound-guided intra-articular prolotherapy and corticosteroid injection both improved pain and functional disability in patients with sacroiliac joint dysfunction. However, prolotherapy provided superior sustained pain relief and greater functional improvement over the 12-week follow-up period. These findings suggest that prolotherapy may be an effective treatment option for patients with SI joint dysfunction.

## References

[1] Yin W, Willard F, Carreiro J, Dreyfuss P (2003). Sensory stimulation-guided sacroiliac joint radiofrequency neurotomy: technique based on neuroanatomy of the dorsal sacral plexus. Spine (Phila Pa 1976).

[2] DePalma MJ, Ketchum JM, Saullo T (2011). What is the source of chronic low back pain and does age play a role?. Pain Med.

[3] Cohen SP, Chen Y, Neufeld NJ (2013). Sacroiliac joint pain: a comprehensive review of epidemiology, diagnosis and treatment. Expert Rev Neurother.

[4] Van Kleef M, Mekhail N, van Zundert J (2009). Evidence‐based guidelines for interventional pain medicine according to clinical diagnoses. Pain Pract.

[5] Cohen SP (2007). Epidemics, evolution, and sacroiliac joint pain. Reg Anesth Pain Med.

[6] Al-Subahi M, Al-Ahaideb A, Al-Saedi A, Alshammari S (2021). Comparative effectiveness of prolotherapy versus corticosteroids in sacroiliac joint dysfunction: a systematic review. Pain Physician.

[7] Patel N (2016). Twelve-month follow-up of a randomized trial assessing cooled radiofrequency denervation as a treatment for sacroiliac region pain. Pain Pract.

[8] Borowsky CD, Fagen G (2008). Sources of sacroiliac region pain: insights gained from a study comparing standard intra-articular injection with a technique combining intra- and peri-articular injection. Arch Phys Med Rehabil.

[9] Soneji N, Bhatia A, Seib R, Tumber P, Dissanayake M, Peng PW (2016). Comparison of fluoroscopy and ultrasound guidance for sacroiliac joint injection in patients with chronic low back pain. Pain Pract.

[10] Hoffman MD, Agnish V (2018). Functional outcome from sacroiliac joint prolotherapy in patients with sacroiliac joint instability. Complement Ther Med.

[11] Mitchell B, Barnard A (2012). Prolotherapy for sacroiliac joint pain. J Sci Med Sport.

[12] Lee JD, Lee DW, Jeong CW, Lee HG, Yoon MH, Kim WM (2009). Effects of intraarticular prolotherapy on sacroiliac joint pain. Korean J Pain.

[13] Buijs E, Visser L, Groen G (2007). Sciatica and the sacroiliac joint: a forgotten concept. Br J Anaesth.

[14] Kim WM, Lee HG, Jeong CW, Kim CM, Yoon MH (2010). A randomized controlled trial of intra-articular prolotherapy versus steroid injection for sacroiliac joint pain. J Altern Complement Med.

[15] Laslett M (2008). Evidence-based diagnosis and treatment of the painful sacroiliac joint. J Man Manip Ther.

[16] Farrar JT, Young JP Jr, LaMoreaux L, Werth JL, Poole RM (2001). Clinical importance of changes in chronic pain intensity measured on an 11-point numerical pain rating scale. Pain.

[17] Fairbank JC, Pynsent PB (2000). The Oswestry Disability Index. Spine (Phila Pa 1976).

